# Implant Mechanics, Biological Milieu, and Peri-Implantitis: A Narrative Review

**DOI:** 10.7759/cureus.67173

**Published:** 2024-08-19

**Authors:** Sarah Mariam, Rajesh Kshirsagar, Shamimul Hasan, Yogesh Khadtare, Komal S Rajpurohit, Himanshi Rai, Devashri Newaskar, Priya Deo

**Affiliations:** 1 Periodontology, Bharati Vidyapeeth (Deemed to be University), Pune, IND; 2 Oral and Maxillofacial Surgery, Bharati Vidyapeeth (Deemed to be University), Pune, IND; 3 Oral Medicine and Radiology, Faculty of Dentistry, Jamia Millia Islamia, New Delhi, IND; 4 Periodontology, Bharati Vidyapeeth Deemed to be University Dental College and Hospital, Pune, IND; 5 Oral Pathology and Microbiology, Bharati Vidyapeeth Deemed to be University Dental College and Hospital, Pune, IND

**Keywords:** peri-implantitis, metagenomics, microbiota, dental abutments, dental implants

## Abstract

Dental implants constitute an important treatment modality for rehabilitating edentulous and partially edentulous arches. With more implant systems in the market, understanding the mechanical aspects of implants is crucial in understanding this indispensable therapy. However, microflora-related factors i.e. biological factors are also crucial. Despite the tremendous success rate of dental implants, it is not averse to failure. Both mechanical and microbial aspects in seclusion or together predispose to implant failure. Newer technological advances have paved the way for advanced techniques to identify the not-so-common flora causing implant failure.

This review focuses on detailed mechanical and biological aspects and the sealing agent used to seal the implant-abutment interface. It also focuses on advanced molecular techniques like metagenomics and transcriptomics. A thorough literature search was performed with selected articles from electronic databases. A combination of in-vivo and in-vitro studies were considered to provide comprehensive information on the subject. Both the biomechanical aspects like micro gap, and microleakage, as well as microbial movements play confluent roles in implant failure.

The focus should be on the different aspects through which microflora can penetrate the inner parts of the implant. Also, newer culture-independent techniques of detecting previously undetected oral flora should be included in future studies.

## Introduction and background

Landmark changes in dental implantology have changed the course of treatment modalities for rehabilitating edentulous and partially edentulous arches. Since their introduction by Branemark in the 1970s, dental implants have become a highly preferred option for rehabilitating missing teeth. Nevertheless, rehabilitation of edentulous and partially edentulous arches is not averse to failure, with previous reports of implant failure in the range of 1% to 19%. These failures can be categorized as early or late depending on the timing of abutment connection: early failures occur before functional loading is applied, whereas late failures occur after occlusal loading or after the first removal of the provisional restoration in cases of immediate implant loading [1].

Various risk factors associated with implant failure are old age, female gender, systemic disorders (diabetes mellitus, cardiovascular disorders, immunodeficiency), chronic smoking, maxillary implant location, adjacent natural dentition, quantity and quality of bone (implant failure is common in osteoporotic patients), poor oral hygiene, implant surface treatments and characteristics, and genetic factors [2].

The risk of implant failure increases with advanced age, as older patients are more likely to experience systemic health changes, have compromised local bone conditions, and may require longer healing times. Smoking impairs the osseointegration process by reducing blood flow due to increased peripheral resistance and platelet aggregation, leading to a lower survival rate for dental implants in smokers. Hyperglycemia can negatively impact the osseointegration of dental implants by altering the response of parathyroid hormone, which regulates phosphorus and calcium metabolism, and by inhibiting osteoblastic differentiation. Osseointegration failure in osteoporotic patients results from a decrease in bone mass and density [1,2].

Esposito et al. stated that the reasons for failure of dental implants include biological failures (biological processes related to bacterial biofilm and some pathogens display catastrophic virulence factors) and mechanical failures (including fractures of implants, coatings, connecting screws, and/or removable and fixed prostheses) [3,4].

**Figure 1:**
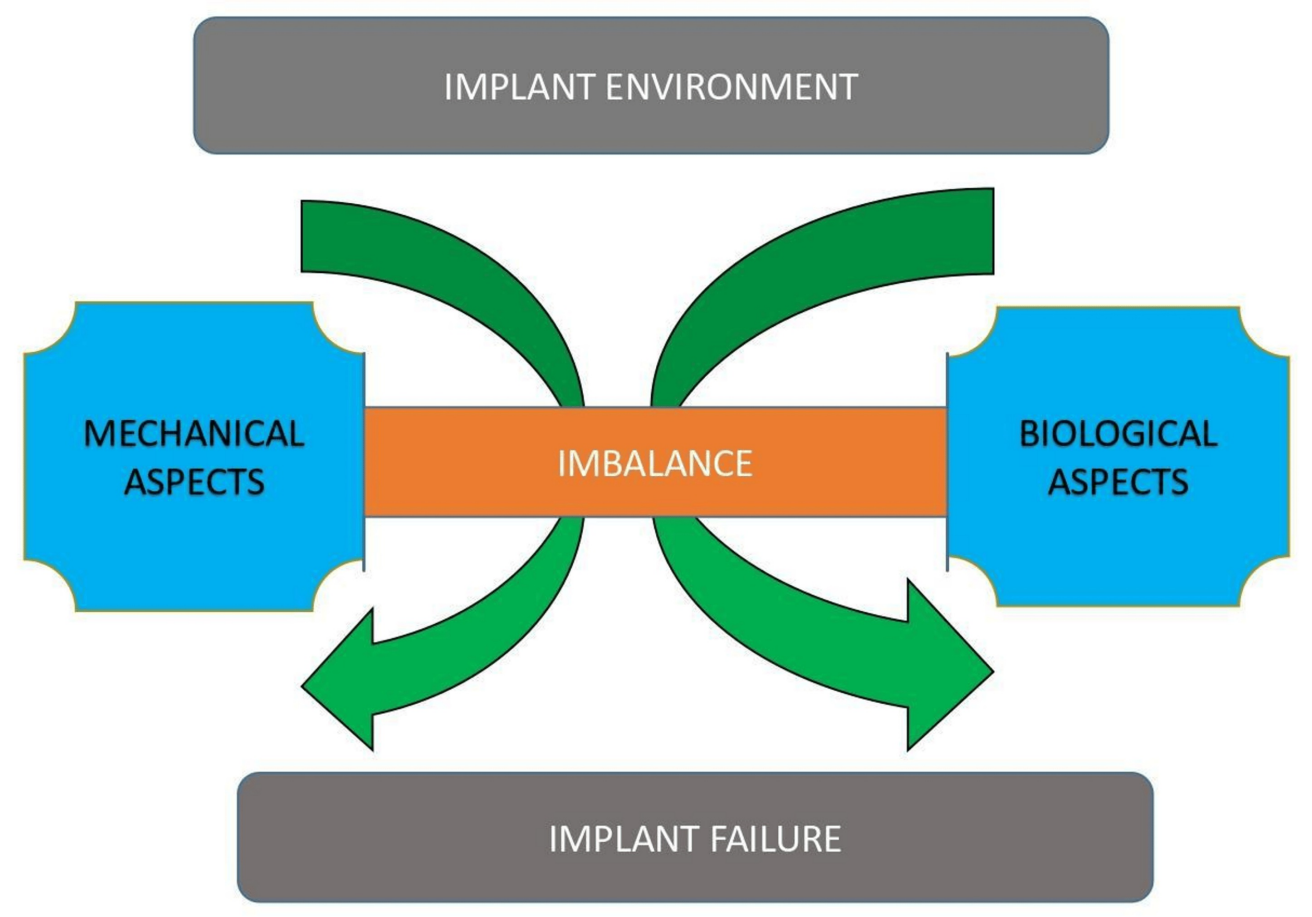
The causes of implant failure. Image credit: Dr. Sarah Mariam

The imbalance in the mechanical and biological factors resulting in implant failure is illustrated in Figure 1.

Periodontitis refers to a chronic inflammatory reaction involving not only superficial gingival tissues but also the periodontal ligament and alveolar bone [5]. According to the American Academy of Periodontology, periodontitis is characterized by symptoms including bleeding on probing, gingival recession, pocket formation, and alveolar bone loss. The condition is primarily driven by periodontal pathogens, with gram-negative bacteria, particularly *Porphyromonas gingivalis*, being central to periodontal diseases. Additionally, periodontitis is an immune-modulatory disorder that involves various inflammatory mediators in its progression [5]. Peri-implantitis is a progressive inflammatory condition affecting the tissues around an osseointegrated implant, resulting in supporting bone loss and implant failure. The condition is characterized by bleeding, pus discharge, increased probing depth, implant mobility, and radiographic evidence of bone loss. The inflammatory process affecting dental implants is more severe and progresses more quickly than the inflammation surrounding a nearby natural tooth [2].

Soft laser irradiation is effective for eliminating bacterial pathogens responsible for peri-implantitis. Systemic antibiotics targeting gram-negative anaerobes modify the microbial composition and clinically improve the condition over a one-year period. Local delivery devices, such as Actisite, which contains fibers with polymeric tetracycline hydrochloride, can significantly reduce the total anaerobic bacterial counts [2]. Additionally, recent research over the past decade has explored advanced antibiofilm techniques, including the use of bacteriophages (viruses that infect bacterial cells), antimicrobial peptides, and photodynamic therapy [6].

The pathophysiology of periodontitis and peri-implantitis is generally directed by the response of the host immune system. Even in a healthy individual, the peri-implant and the periodontal tissues that are in proximity of dental biofilm, showcase an active immune response [6].

A chain of complex low-grade inflammation follows, and it involves both acquired and innate immunity along with the complement system [7,8]. An imbalance in inflammatory mediator production due to dysbiosis results in the release of toxic products by host cells. Excessive production of toxins from periodontal pathogens destroys the surrounding soft tissues [9]. To endure the persistent contact with microbes and their virulence factors in the peri-implant and periodontal pockets, the host frames the expression of defense mediators.

Initial identification of bacteria or virulence factors by resident cells occurs through communication between the TLR (toll-like receptors) and bacteria, and this accelerates the presentation of chemokines. Later on, E-selectin and intercellular adhesion molecules are produced at the surface of local endothelial cells. These molecules prompt the migration of polymorphonuclear neutrophils (PMNs) from the gingival vessels to the junctional epithelium, where they function as the initial defense mechanism in the peri-implant mucosa and periodontium [10].

Once the connective tissue is invaded, components of the extracellular matrix like proteoglycans, collagens, etc. are also involved in peri-implantitis and periodontitis. This dismantling of the extracellular matrix is performed by a group of enzymes including collagenases, and matrix metalloproteinases. They are released locally by PMNs or macrophages or resident tissue cells like gingival fibroblasts. These inflammatory and immune responses that lead to peri-implant and periodontal diseases are initiated by microorganisms forming biofilms on enamel and titanium surfaces, which closely interact with the host's junctional and sulcular epithelium [11].

**Figure 2:**
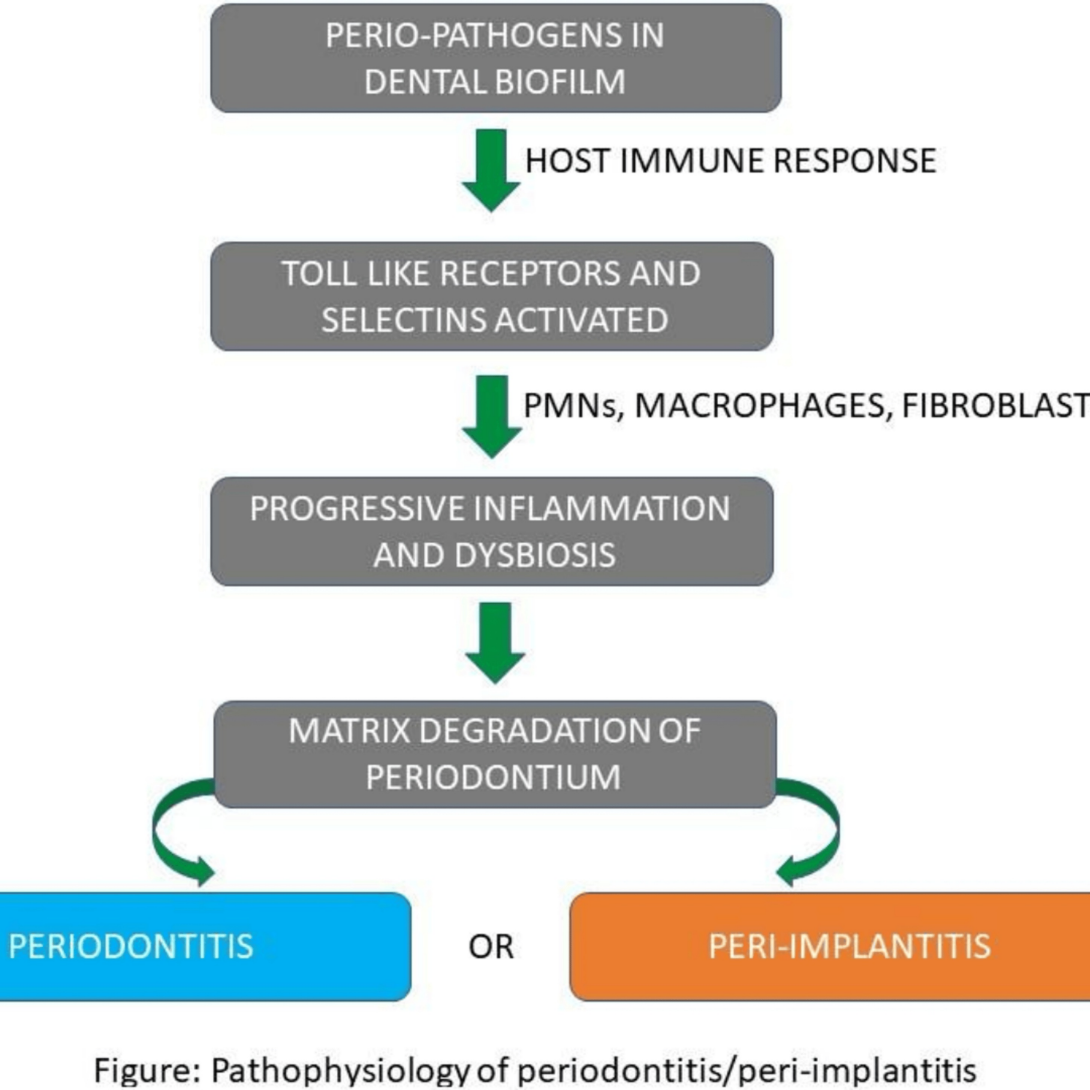
Pathophysiology of periodontitis and peri-implantitis. Image credit: Dr. Sarah Mariam

The pathophysiology of periodontitis and peri-implantitis is illustrated in Figure 2.

The pathogenicity of oral microbes is influenced by the biofilm of dental plaque. Factors that may favor bacterial overgrowth around the implant include pre-existing periodontal disease, patient hygiene, design of the implant, and implant abutment interface [12]. Biofilms consist of microbial communities that adhere to both biological and non-biological surfaces, with protection provided by an extracellular polymeric matrix [13].

It is believed that biofilm-forming microorganisms are responsible for the majority of microbial infections, accounting for about 65%. This poses a major public health challenge due to their capacity to evade the host immune system and resist most antimicrobial treatments, resulting in prolonged infections [14].

For accurately characterizing various pathogens in a biofilm, molecular methods are the best options. A plethora of knowledge gained from conventional methods (culture, microbial staining, biochemical tests, immunological methods) and advanced microbiological methods (genomics, and transcriptomics) has improved our understanding of peri-implant microflora immensely. Microflora is intimately related to the development of peri-implant disease [15].

We can infer that the mechanical components of implants have a role to play in forming various bacterial niches that can shift the balance in favor of peri-implantitis and subsequent implant failure.

## Review

Recent research has focused on mechanical factors like micro gap, microleakage, implant screw, implant screw abutment connection, preload, sealing agent, torque, and biological factors such as oral microbiome. Our research confluences the role of these factors and their possible links with peri-implantitis and implant failure. The research also discusses next-generation sequencing techniques, metagenomics, transcriptomics, and its relationship with oral microbiome.

Search strategy

A comprehensive literature review search was carried out using electronic databases such as PubMed, Scopus, Web of Science, Embase, and Google Scholar. The search included keywords such as dental implants, dental abutments, oral microbiota, implant failure, peri-implantitis, and metagenomics. Included study considered case reports/case series, review articles, case-control, cross-sectional, retrospective/prospective cohort studies published up to January 2024 in the English language which analyzed the possible relationship between implant biomechanics, microflora, and implant failure. Only studies with a sizable sample size, structured research design, and definite conclusions were considered. Low-evidence articles in non-indexed journals with a smaller sample size were excluded.

Mechanical aspects

Implant abutment connection is a critical aspect of implant geometry. This interface is influenced by critical factors like hex design (external hex, internal hex, platform switch), screw design and its cross-section, screw diameter, preload, and after-load [16]. Fracture of the abutment screw can be due to misfit and lack of acquiescent adaptation between abutment and prosthesis. The internal hex is better than the external hex due to the lengthy inner wall that minimizes micro-movements. It also can dissipate lateral forces, has a more stable connection, and improves force distribution [17]. However, an apparent disadvantage of internal hex is that thinner inner walls may lead to strain at the cervical area. This stress shift could be an implicating factor in marginal bone resorption or even implant fracture [18].

Type of Implant

It is recommended to place implants as early as possible, with a focus on achieving the greatest possible length. Longer implants are thought to offer a higher survival rate and a better prognosis [19]. Recent studies have shown that short implants are as effective as implants of ≥10 mm in length for both fully and partially edentulous patients [20,21].

Implant Surface

The surface of dental implants is critical in influencing bone-implant contact and the rate of bone apposition around the implants [21]. There are two types of implant surfaces: Hydrophilic and hydrophobic surfaces. Hydrophilic surfaces, unlike hydrophobic ones, enhance interactions with biological fluids and cells, leading to improved surface wettability. Implant surfaces with identical chemical compositions can exhibit different contact angles for biological fluids depending on their topography. Rough surfaces, such as those that are sandblasted and etched, tend to be more wettable compared to smoother surfaces [22].

Implant Screw

Ideally, the thread diameter should be narrower than the head of screw. Flat-headed screws and abutments are preferred as it redistributes forces evenly within the body of the fixture. To reduce the tensile forces in threads a tapered head design should be used [23].

Screw Material

Screw material can either be of gold or titanium. Since the elastic modulus of gold is higher than titanium it allows slight micro movements to redistribute forces to the threads. However, they are more prone to fracture [24]. The prime drawback of titanium screws is caused by the frictional phenomenon called “galling”. It is a type of adhesive wear mechanism causing minor changes to retaining screw threads and implant bodies [25].

Preload

Screw tightening produces a unique force inside the implant screw called the preload [26]. Loss of preload accounts as one of the major causes of screw loosening. Preload is the axial force applied in the screw neck, located between the first mating thread and the abutment screw head. This tensile force ensures that the abutment is firmly attached to the implant. The relationship between applied torque and preload depends on several factors including screw geometry, surface texture, material properties, lubrication level, tightening speed, and joint integrity [27]. Adequate preload leads to increased fatigue resistance, decreased micromotion of the abutment screw interface, and fewer chances of loosening the screw [28].

Micro Gap

The micro gap definition is the microscopic space existing between implant-abutment interfaces [29]. An accurate match between implant and abutment is not possible due to precision limitations during the production process [30]. According to the literature, castable abutments exhibit larger micro gaps than machined abutments [31]. Categories of abutment connections are internal hex, external hex, conical, and platform switch. Butt joint connection has lower sealing efficacy than the Morse taper connection [32]. Thus, the degree of fit of the implant abutment interface primarily deals with the degree of taper and connecting area. Tightening force is larger than removing force when the taper degree is larger than 5.8 degrees [33]. On the other hand, smaller taper results in tighter connections. Most connections require a screw with preloaded force to achieve close approximation with the implant-abutment interface. However, an exception to this standardization is a 1.5-degree Morse taper connection having a sizeable surface area of abutment and implant [34,35].

Sealing Agent

In dental implants, sealing agent refers to material placed in implant abutment screw holes. Whatever material we use as a sealing agent, it has a definitive role in peri-implant health considering the phenomenon of micro-leakage at junctions. This phenomenon is exacerbated when the implant is loaded [36]. Though the indication for cotton is that it is to be used under a temporary restoration, it is routinely used as a sealing agent in implant abutment screw holes. Also considering studies which suggest that cotton has the highest concentration of microbial count, thus, creating a milieu conducive to the multiplication of anaerobic bacteria, polyvinyl silicone and polytetrafluoroethylene provide options other than cotton. Recent studies have proved the colonization and multiplication of oral flora inside the fixture of the implant. Reduction in bacterial load inside the implant cavity reduces inflammation around the implant and subsequent peri-implant bone loss [37].

Further research is needed to assess the potential of the many sealing agents for reducing microbial leakage, microbial colonization, and multiplication of the oral bacteria in the abutment and internal part of the implant. Longitudinal studies are required to assess the effect of various sealing materials on the oral microbial profile and the sequel of microleakage on the long-term success of peri-implant health.

Microleakage

Considering two-piece implant arrangements, the micro gap size of the two components ranges from 0.1 microns to 10 microns before load application. This may increase after cyclical loading. The size of most oral bacterial flora is in the range of 0.2-1.5 microns and a length of around 10 micrometers [38]. This creates a nidus of entry of bacterial products and endotoxins to pass freely and enter the internal surface of the implant and the peri-implant area [39]. The reaction of this response i.e. inflammation has been confirmed by the infiltration of neutrophils near implant-abutment interface irrespective of the position of the implant [40].

Microleakage and bacterial penetration can vary in different implant systems. This difference could be because of the different taper degrees and connecting areas. One study evaluated implant-abutment contact areas and micro gaps by X-ray three-dimensional microtomography at the interface of abutments in varied types of connections like screw retained internal hex, Morse cone taper internal connection, screwed trilobed connection.

Results concluded that various voids and gaps were present in screw-retained prostheses. In internal connections (Morse taper), no detectable gaps were found and there was congruity between abutment and implant. The study concluded that observed differences in bacterial penetration may be due to different types of implant-abutment joints [41].

However, a study was done to scrutinize the in-vitro capability of four Morse taper system units to hamper the penetration of bacteria through the interface of abutment and implant. Implants with abutments were dipped in Streptococcus sanguinis broth and subsequently observed by scanning electron microscopy (SEM) for micro gap size, bacteria, and internal surfaces. The study concluded that the sealing capability at the implant-abutment interfaces of commercial Morse taper units was not proficient enough to protect it from bacterial penetration. Also, a low microleakage level of bacteria corresponds to a large torque value [42].

Torque

The force used to insert a dental implant is called insertion torque, which measures the amount of torque required to advance the implant into the prepared osteotomy. Besides indicating bone quality, insertion torque is essential for assessing the implant's primary stability and determining the loading protocol, both of which are critical for the implant's success. Higher insertion torque usually leads to better primary stability, whereas lower values are often associated with higher failure rates. This measurement is generally expressed in Newton centimeters (Ncm). Studies suggest that an optimal insertion torque of around 35 Ncm is considered ideal [43].

Factors that affect insertion torque include bone density, bone hardness, the use of undersized drills, and tapered implant design. Insertion torque is directly proportional to bone density, being highest in D-1 type bone and lowest in D-4 type bone unless compression techniques are employed to enhance stability. Employing undersized drills and implants with a tapered design can create local compression, improving stability. Using a drill that is much smaller in diameter than the implant may initially boost primary stability but can also cause greater necrotic bone and remodeling, resulting in decreased stability until new bone forms. On the other hand, using a larger diameter drill generally leads to lower initial stability but reduces necrotic bone and remodeling, promoting prolonged primary stability and quicker secondary stability through faster-woven bone formation. Tapered implants create greater compression than parallel implants during insertion because they apply lateral bone compression along their entire length. This leads to a progressively increasing insertion torque. Consequently, the stresses are distributed evenly over the entire surface of the implant, rather than being focused on a few localized spots [43].

During the insertion of implants into the bone tissue and the application of the torque to obtain the initial stability, the sets (implant and abutment) are subjected to torsional forces, which depending on their intensity can affect the structure of these parts. Torquing an implant abutment screw refers to the application of rotational force to tighten the screw that connects the dental implant fixture to the abutment. The screw's helical thread allows it to move vertically within the implant as it rotates, tightening or loosening based on the direction of rotation [44].

Occlusal Overloading

Occlusal overloading, evident through mechanical complications, is a major cause of biomechanical implant issues. It can also impair the delicate connection between the bone and implant surface, resulting in peri-implant bone loss and possible implant failure [45]. The precise mechanism of peri-implant bone loss resulting from occlusal overloading remains uncertain due to various confounding factors. Nevertheless, it is evident that occlusal overloading is positively correlated with peri-implant marginal bone loss [46].

**Figure 3:**
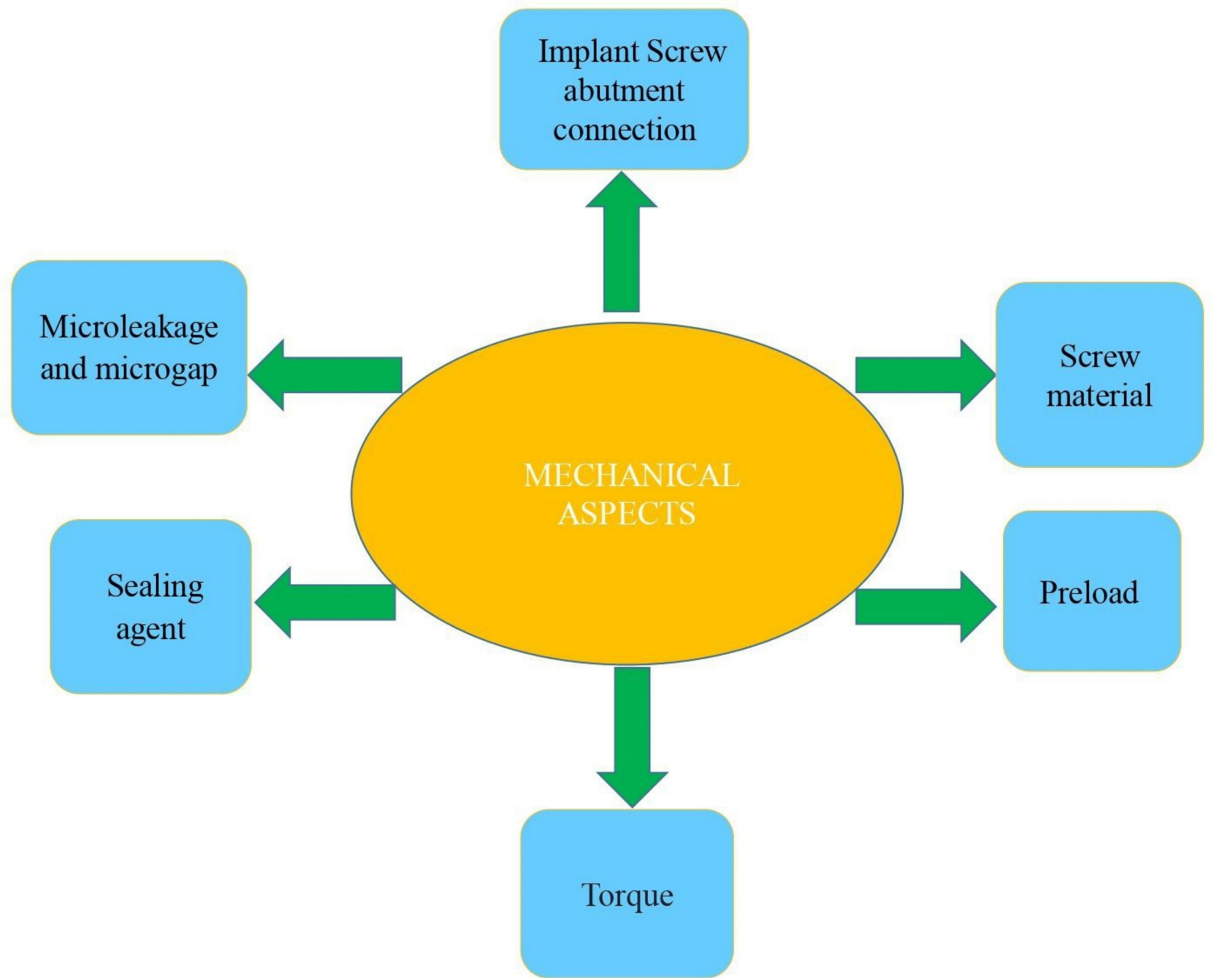
Depicting the mechanical aspects of implant failure. Image credit: Dr. Sarah Mariam

Various mechanical factors associated with implant failure are depicted in Figure 3.

Biological aspects

The revolutionary advances around implants cannot change the fact that it has provided oral tissues with a new artificial milieu, prone to the development of oral biofilms. These biofilms can lead to inflammatory destruction of the periodontal tissues around the dental implant, called peri-implantitis. The flora of periodontal microbial infections resembles that of peri-implantitis. However microbial difference between these two clinical entities is likely to increase with the application of microbial genomics and transcriptomics [47].

Oral Microbiome and DNA Sequencing Approaches

Metagenomics is the direct analysis of genetic makeup contained within a biological sample. It provides vital information about the gene composition at the functional level of microbial communities providing a comprehensive description [48].

Recent literature reveals that metagenomics analysis in healthy and disease conditions showed microbial signatures for peri-implant disease defined as the peri-implantitis-related complex containing seven of the most impactful bacteria. *Fusobacterium nucleatum* acts as a keystone pathogen and is intricately related to mucositis. Analysis showed high accuracy in diagnosis and prognosis for peri-implant diseases. This study identified plaque-related microbiome markers related to peri-implant diseases and their severity [49].

Both periodontitis and peri-implantitis are polymicrobial diseases caused by subgingival plaque accumulation. There are only a few studies related to microbiota and genomics of peri-implantitis. A study analyzed peri-implantitis and periodontitis with a sample size of 21 patients through metagenomics and data related to meta-transcriptomics from 12 patients were obtained from a database. Species specific to peri-implantitis in the co-occurrence network were *Solobacterium moorei *and *Prevotella denticola*. The role of specific receptors of plasmin was higher in peri-implantitis [50].

Using a 16S rRNA analysis microbiota in peri-implantitis was diverse and included gram-negative species. Phyla associated with peri-implantitis included *Chloroflexi, Peptostreptococcus stomatis, Tenericutes, Synergistetes, Parvimonas micra, *and *Solobacterium moorei*. Low levels of periodontitis-causing bacteria, like *Aggregatibacter actinomycetemcomitans* and *Porphyromonas gingivalis,* were observed around peri-implant bone defects. The study concluded that peri-implantitis was associated with a more complex microbiota in comparison to chronic periodontitis and healthy patients [51].

A recent study concluded that the microbiome at peri-implantitis sites exhibited variations from that of healthy implant sites in both taxonomic and functional aspects. Additionally, the presence of periodontitis affected the dominant species in peri-implantitis sites. *Prevotella spp.* and *P. endodontalis* displayed notable differences in the peri-implantitis groups across various periodontal conditions. Nevertheless, *T. forsythia, P. gingivalis, T. denticola, *and *P. endodontalis* remained consistently associated with peri-implantitis and inflammatory clinical parameters, regardless of the presence of periodontitis. The primary functional difference between diseased and healthy implants pertains to flagellar assembly, which is critical for the invasion of epithelial cells [52].

Oral Microbiome and Transcriptomics

Transcriptomics microbiological analysis utilizes methods to know about an organism’s transcriptome which is the aggregate of all its RNA transcripts logs. This information subset is stored and maintained in the DNA of its genome and protein expression is evaluated through transcription. A transcriptome in a snapshot of time evaluates the total transcripts present in a cellular structure [53].

In a published study, ten healthy and ten compromised patients’ peri-implant tissues were included and subjected to a transcriptome-wide microarray profiling process covering over 20,000 genes. Pathways in peri-implant health and disease were discovered and differential expression of genes was evaluated. The study concluded that all peri-implant samples had highly transcribed cellular respiration pathways implicated in oxidative stress, indicating that implant-specific variables might induce a persistent state of oxidative stress [54].

A recent cross-sectional study involved RNA sequencing and analysis on gingival samples taken from healthy, periodontitis, and peri-implantitis patients. The study concluded that although periodontitis and peri-implantitis showed common gene expression that was clearly differentiated from healthy conditions, there were also unique gene patterns that were differentially expressed only in peri-implantitis [55].

**Figure 4:**
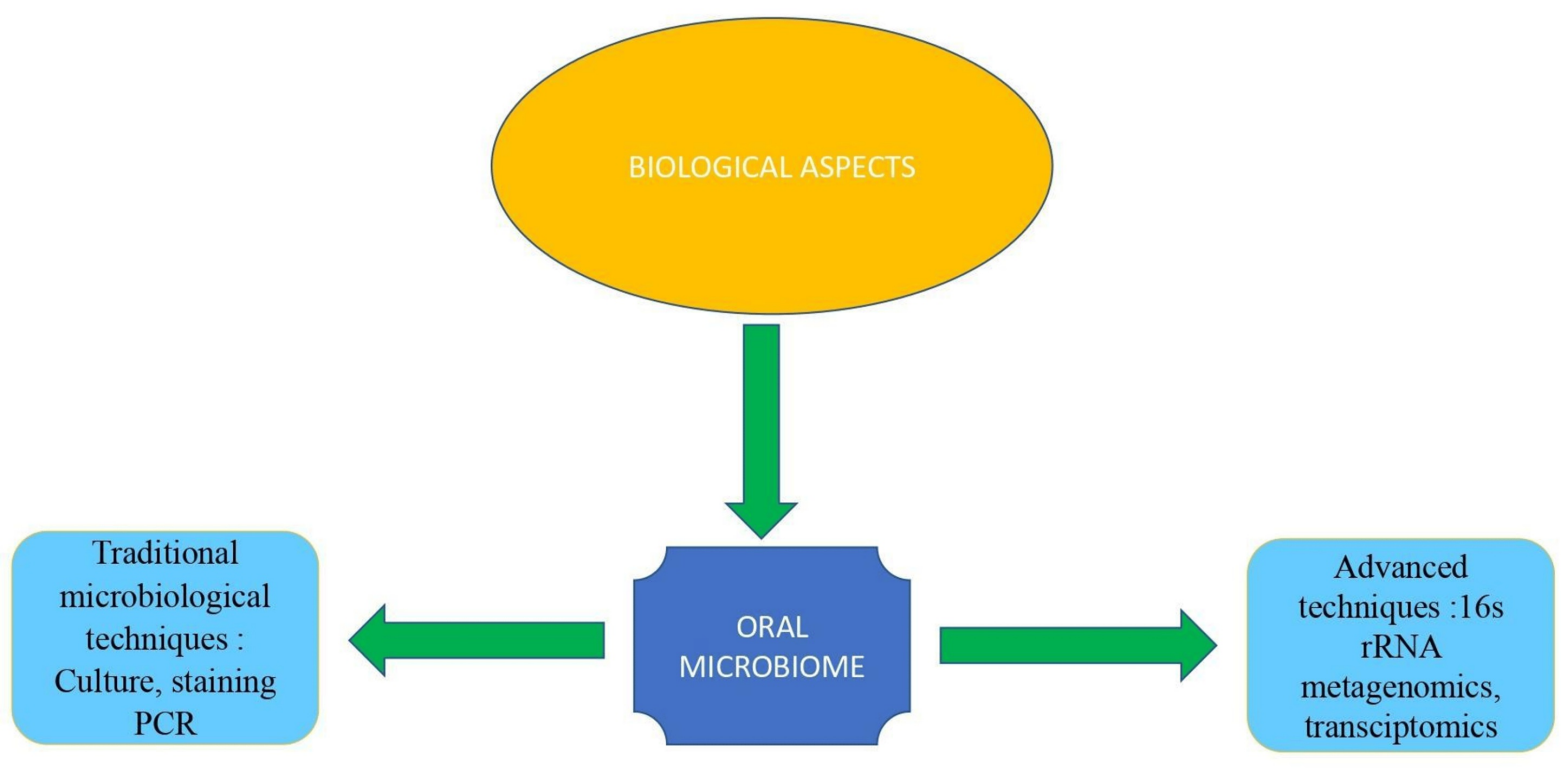
Depicting the biological aspects of implant failure. Image credit: Dr. Sarah Mariam

Bacterial biofilms and their diagnostic methods are depicted in Figure 4.

## Conclusions

Various biological and mechanical factors are indispensable to the success of implants. This article summarizes various mechanical factors like the type of screw material, sealing agent, preload, implant torque, and micro-gap in defining implant dynamics. It also discusses various biological factors like bacteria causing periodontitis and peri-implantitis, microleakage around various implant abutment interfaces, oral microbiome, and advanced techniques of metagenomics. Selecting an ideal material is paramount to avoid any unforeseen clinical developments.
